# Effects of Antirheumatic Treatment on Cell Cholesterol Efflux and Loading Capacity of Serum Lipoproteins in Spondylarthropathies

**DOI:** 10.3390/jcm11247330

**Published:** 2022-12-09

**Authors:** Ingrid Hokstad, Daniela Greco, Gia Deyab, Morten Wang Fagerland, Stefan Agewall, Gunnbjørg Hjeltnes, Francesca Zimetti, Franco Bernini, Nicoletta Ronda, Ivana Hollan

**Affiliations:** 1 Lillehammer Hospital for Rheumatic Diseases, 2609 Lillehammer, Norway; 2 Institute of Clinical Sciences, University of Oslo, 0318 Oslo, Norway; 3 Department of Food and Drug, University of Parma, 43124 Parma, Italy; 4 Department of Laboratory Medicine, Vestre Viken Trust, 3004 Drammen, Norway; 5 Oslo Centre for Biostatistics and Epidemiology, Research Support Services, Oslo University Hospital, 0424 Oslo, Norway; 6 Department of Medicine, Oslo University Hospital Ullevål, 0424 Oslo, Norway; 7 Department of Internal Medicine, Innlandet Hospital Trust, 2819 Lillehammer, Norway; 8 Beitostølen Health and Sport Centre, 2953 Beitostølen, Norway; 9 Institute for Health Sciences, The Norwegian University of Science and Technology, 7034 Gjøvik, Norway

**Keywords:** spondyloarthropathies, psoriatic arthritis, anchylosing spondylitis, psoriatic arthritis, anti-rheumatic treatment, lipoproteins, high density lipoprotein, low density lipoprotein, cell cholesterol efflux, macrophages

## Abstract

Spondyloarthropathies (SpA) are associated with increased cardiovascular risk. Among possible mechanisms is the dysfunction of serum lipoproteins in regulating cell cholesterol homeostasis. Cholesterol efflux capacity (CEC)—the atheroprotective ability of HDL (high density lipoproteins) to accept cholesterol from macrophages—might predict cardiovascular disease independently of HDL-cholesterol levels. We aimed at evaluating modifications of CEC and of the atherogenic cholesterol loading capacity (CLC) of serum lipoproteins in psoriatic arthritis (PsA) and ankylosing spondylitis (AS) following anti-rheumatic treatment. A total of 62 SpA patients (37 PsA and 25 AS) were evaluated before and after treatment with tumor necrosis factor inhibitor and/or methotrexate. CEC and CLC were measured by radioisotopic and fluorometric techniques, respectively. Endothelial function was assessed by finger plethysmography (Endopat). In the whole SpA group, total and HDL-cholesterol increased after treatment, while lipoprotein(a) decreased and CLC was unchanged. Treatment was associated with increased Scavenger Receptor class B type I (SR-BI)-mediated CEC in the AS group. SR-BI- and ABCG1-mediated CEC were negatively associated with inflammatory parameters and positively related to coffee consumption. SR-BI CEC and CLC were positively and negatively associated with endothelial function, respectively. Our pilot study suggests that anti-rheumatic treatment is associated with favorable modulation of lipoprotein quality and function in SpA, particularly in AS, in spite of the induced increase in total cholesterol levels. If confirmed in a larger population, this might represent an atheroprotective benefit beyond what is reflected by conventional serum lipid profile.

## 1. Introduction

Spondyloarthropathies (SpA) are associated with increased risk of cardiovascular disease (CVD) [1,2,3]. The exact mechanisms linking SpA and CVD remain unclarified, but appear to include traditional cardiovascular (CV) risk factors, medication side effects (e.g., NSAIDs), and SpA specific factors, such as inflammatory processes.

How lipid metabolism affects CV risk in autoimmune and rheumatologic conditions is a complex question. In fact, inflammation may be associated with modifications not only of lipoprotein levels, but also of their composition and function. For example, patients with active chronic inflammatory diseases, such as SpA and RA, often display low serum levels of total cholesterol (TC) and low density lipoprotein cholesterol (LDL-C) together with increased CV risk, a phenomenon referred to as “the lipid paradox” [4]. Moreover, high density lipoproteins (HDL)—traditionally considered a lipoprotein with atheroprotective properties—can acquire proinflammatory and proatherogenic properties under inflammatory conditions [5]. Although countless epidemiological studies have found high HDL-cholesterol (HDL-C) to be associated with reduced CV risk, drugs increasing HDL-C, or heritable traits associated with high HDL-C, do not necessarily reduce CV morbidity and mortality, underlining the importance of evaluating HDL function, not just serum levels [6,7].

Cholesterol efflux capacity is a measure of HDL’s ability to remove excess cholesterol from peripheral cells, the first step in reverse cholesterol transport. Cholesterol efflux from macrophages is mediated mainly via three receptor-mediated pathways: ATP-binding cassette transporter A1 and G1 (ABCA1 and ABCG1), and scavenger receptor BI (SR-BI) [8]. Whereas the relationship between serum HDL-C and CVD is inconclusive, CEC appears to be inversely associated with atherosclerotic CVD, carotid intima-media thickness, and coronary plaque burden [9,10,11]. Furthermore, CEC seems to predict CVD independently of C-reactive protein (CRP), HDL-C, coronary calcium, and family history of CVD [12]. Cholesterol loading capacity (CLC), on the other hand, reflects the ability of patients’ sera to induce cholesterol loading in macrophages, and may be increased in conditions with altered LDL metabolism [13].

Inflammation appears to impact HDL quality and function [14,15], and CEC has been found to be altered in various rheumatologic autoimmune diseases [16,17,18]. A few studies have reported reduced CEC in psoriasis and SpA patients compared to controls [19,20,21]. In these reports, CEC was evaluated as total cell cholesterol efflux; with no information on CEC specific pathways, which might provide supplemental information about HDL quality and maturation.

Knowledge about how anti-rheumatic treatment influences CEC is very limited, with some studies showing reduced or unchanged CEC following treatment [21,22], and others demonstrating increased post-treatment CEC [23,24,25]. The aim of this study was to investigate CEC, separately for each of the three main pathways of cell cholesterol efflux, and CLC in SpA patients (psoriatic arthritis, PsA, and anchylosing spondylitis, AS), before and after treatment with methotrexate (MTX) and/or tumor necrosis factor inhibitors (TNFi). We further searched for associations of CEC and CLC with CV risk factors (including lipid levels, HbA1c and endothelial function), lifestyle factors, and SpA characteristics.

## 2. Materials and Methods

### 2.1. Study Design and Participants

We studied 62 patients (37 PsA and 25 AS) from the PSARA (PSoriatic arthritis, Ankylosing spondylitis, Rheumatoid Arthritis) study, for which serum samples were available for the lipoprotein function evaluation. The study was conducted according to the guidelines of the Declaration of Helsinki, was approved by The Norwegian Regional Ethical Committee, and was registered in the Norwegian Biobank register, and “Clinical Trials” (NCT00902005). Written informed consent was obtained from all subjects involved in the study.

The PSARA study prospectively enrolled PsA patients who fulfilled the Caspar criteria and AS patients who met the modified New York criteria. A rheumatologist not involved in the study determined appropriate treatment according to the national treatment recommendations and clinical judgment. Of the 62 patients included in the present study, 34 patients were started on TNFi, 18 on MTX, and 10 on a combination of TNFi + MTX. Patients were examined at baseline, after 6 weeks, and after 6 months of treatment. Assessment included information about demographic data, lifestyle, medication, and medical history (including SpA characteristics and CV risk factors), physical examination, blood analyses, and endothelial function measurements. As PsA and AS showed significant differences both in serum lipid profile at baseline and following treatment, we analyzed the data relative to the two diseases separately.

### 2.2. Endothelial Function

To evaluate endothelial function, we used finger plethysmography (Endopat 2000, Itamar), which measures pulsatile arterial volume changes in fingers before and after induction of reactive hyperemia. A reactive hyperemia index (RHI) below 1.67 was defined as endothelial dysfunction, in line with the manufacturer’s recommendations.

### 2.3. Cell Cholesterol Transport Parameters

#### 2.3.1. Preparation of apoB-Depleted Serum

Precipitation of serum apoB-containing lipoproteins was obtained by adding 40 parts polyethylene glycol (PEG) solution (20% PEG in 200 mM glycine buffer, pH 7.4) to 100 parts of serum [26]. After a 20 min incubation, the precipitate was spun down by high-speed centrifugation (10,000 rpm, 30 min, 4 °C) and the supernatant containing the HDL lipoprotein fraction (apoB-depleted serum) was collected, for the measurement of CEC.

#### 2.3.2. HDL Cholesterol Efflux Capacity Measurement

CEC mediated by specific pathways was measured using adequate cell models [27] by analyses in triplicate. For SR-BI mediated CEC, Fu5AH rat hepatoma cells were labelled for 24 h with [1,2–3H]-cholesterol (PerkinElmer, Milano, Italy) with 1% FCS and 2 µg/mL of an ACAT inhibitor (Sandoz 58035, Sigma-Aldrich, Milano, Italy) to ensure that all labelled cholesterol remained as free cholesterol. Cells were then treated or not with 10 µM Block Lipid Transfer-1 (BLT), which inhibits SR-BI [28], before the incubation with 2.8% apoB-depleted patient serum for 4 h. The SR-BI specific contribution to cholesterol efflux was calculated as the difference between percentage efflux obtained in BLT-1 treated and untreated cells. For ABCG1-mediated CEC, Chinese hamster ovary cells transfected and not transfected with the human ABCG1 gene were used. After labelling for 24 h with 1 μCi/mL [1,2–3H]-cholesterol in presence of 10% FCS, cells were treated for 6 h with 1.4% apoB-depleted patient serum. The specific ABCG1-mediated cholesterol efflux contribution was calculated as the difference between CEC obtained in ABCG1-transfected and non-transfected cells. To measure ABCA1-mediated CEC, J774 murine macrophages were labelled with 2 µCi/mL [1,2–3H] cholesterol in presence of 1% FCS and 2 μg/mL of an ACAT inhibitor (Sandoz 58035, Sigma-Aldrich, Milano, Italy) for 24 h. Cells were then incubated or not with 0.3 mM of a cAMP analogue (cpt-cAMP; Sigma-Aldrich, Milano, Italy), which induces ABCA1 expression [29], before the incubation with 2.4% apoB-depleted patient serum for 4 h. The specific ABCA1-mediated efflux contribution was calculated as the difference between cholesterol efflux in cAMP-treated versus non-treated cells.

HDL CEC values were expressed as a percentage of the radioactivity released into the medium over the total radioactivity incorporated by cells. The HDL fraction of a standard serum obtained from a pool of normolipidemic subjects, native plasma HDL isolated from healthy donors by ultracentrifugation [30], and lipid-free human Apolipoprotein A-I (apoAI) (Sigma-Aldrich) were tested in each assay and their CEC values were used to normalize the patient samples values obtained in different experiments, to correct for the inter-assay variability. A second pool of human normolipidaemic sera was tested in each assay and its CEC, after normalization, was the index of the intra-assay variability.

#### 2.3.3. Serum Cholesterol Loading Capacity (CLC) Measurement

THP-1 cells were seeded in 24-well plates in the presence of 100 ng/mL Phorbol 12-Myristate 13-Acetate (Sigma Aldrich, St. Louis, MO, USA) for 72 h to allow differentiation into macrophages. Cells were incubated with 5% lipoprotein deficient serum (LPDS, Sigma-Aldrich) for 24 h and subsequently exposed to 10% (*v*/*v*) whole serum collected from patients, in triplicate, for 24 h. At the end of incubation, cell monolayers were lysed in 1% sodium cholate solution (Sigma-Aldrich) supplemented with 10 U/mL DNase (Sigma-Aldrich). Cholesterol was measured fluorometrically using the Amplex Red Cholesterol Assay Kit (Molecular Probes, Eugene, OR) following manufacturer’s instructions, as previously described [26]. An aliquot of the cell lysates was taken to measure cell protein by a modified Lowry method. CLC was defined as macrophage cholesterol content in the cell extract after exposure of cells to serum and expressed as micrograms of cholesterol per micrograms DNA. DNA content in the cell lysates was measured through the deoxyribose-diphenylamine reaction method [31]. To check for adequate cell responsiveness, sera obtained from pools of normolipidemic and hypercholesterolemic subjects were tested together with serum samples in each assay. The relative CLC values were used to normalize the different experiments to correct for inter-assay variability.

### 2.4. Statistics

We included all participants in our statistical analyses, also non-completers. For normally and non-normally distributed variables the baseline characteristics are presented as mean and standard deviation (SD) or median and interquartile range (IQR), respectively. For continuous variables, the Mann–Whitney U test was applied for comparison between the treatment groups, and Wilcoxon signed rank test for comparisons within a group. Categorical variables between groups were compared by the chi-squares test or the Fisher mid-P test, based on Cochran’s criterion [32].

We performed simple regression analyses to assess associations between CEC or CLC at the various time points and demographic data (age, gender), inflammatory activity (C-reactive protein (CRP), white blood cells (WBC), erythrocyte sedimentation rate (ESR), hemoglobin (Hb), disease activity scores (BASDAI, MHAQ, BASFI, BASMI, patient and physician global assessment), and indicators of CV risk (established CVD, body mass index (BMI), smoking status, alcohol consumption, exercise, Reactive Hyperemia Index (RHI), co-medication (statins, NSAIDS, acetylsalicylic acid, coffee intake (self-reported number of daily cups, stratified into three groups; 0–4 cups, 5–10 cups, or >10 cups), glucocorticoids, ACE-inhibitors) and serum levels of TC, LDL-C, HDL-C, lipoprotein a (Lp(a)), triglycerides (TG), HbA1c and homocysteine. For these analyses, data from 62 patients were available. In multiple linear regression analyses, we included variables with a *p*-value < 0.10 in univariate analyses. We then ran adjusted models with variables showing a consistent relationship with the parameter of interest, where each significant variable was corrected for age, sex, CRP, ESR, HDL-C and LDL-C. The PSARA study was designed as a database intended to explore several topics. Thus, our study was not necessarily powered to detect clinically meaningful treatment effects and associations but could be considered more of a convenience sample. Furthermore, *p*-values < 0.05 were considered statistically significant, and all tests were two-tailed. We used Prism 7 and SPSS 24 softwares.

## 3. Results

### 3.1. Baseline Demographics and Laboratory or Instrumental Parameters

Patient characteristics at baseline are shown in Table 1. PsA and AS patients did not differ with respect to age, inflammatory markers, or disease activity scores, while gender distribution differed between the groups. All AS patients were treated with TNFi, while PsA patients received TNFi, MTX, or both. Considering CV risk factors, AS patients had less favorable risk profiles with respect to current smoking habits. For co-medication, the only significant difference regarded statins, used by about one third of AS patients and by only two PsA patients. Serum lipid profile differed in that TC was significantly higher in the PsA group, but if adjusting for statin use, there were no significant baseline differences in either TC, HDL-C, LDL-C or Lp(a) between AS and PsA patients. Further, there were no significant baseline differences in CEC or CLC in statin users compared to non-users. Baseline TC and LDL-C were however significantly lower in statin users, while there were no significant differences in HDL-C and Lp(a) between patients on statins and those without such treatment. With respect to lipoprotein function parameters, CEC did not differ between AS and PsA patients in either of the three pathways, but CLC was significantly higher in PsA patients. When adjusting for statin use, CLC was not significantly different between AS and PsA patients.

### 3.2. Changes in Lipid Parameters following Anti-Rheumatic Treatment

In the total SpA group, no significant changes in CEC pathways or in CLC were observed at any time point after treatment. A trend towards increased SR-BI CEC was however observed after 6 months of therapy. Serum lipid profile showed a significant increase for TC, HDL-Cand apoA1 after 6 weeks, lasting for 6 months (Table 2). Lp(a) was significantly decreased after 6 weeks, and after 6 months (Table 2).

Analyzing the two diseases separately, no changes in CEC or CLC were found over time in PsA patients, while Lp(a) decreased, and ApoA-I increased after 6 weeks (Table 3). In AS, SR-BI mediated CEC was significantly increased after 6 weeks but not after 6 months. These changes in SR-BI mediated CEC were not independent of increase in HDL-C during the same time period. Serum lipid profile showed TC increase at both time points after treatment and increase in LDL-C after 6 weeks (Table 3). HDL-C and apoA1 were also increased at both time points. Lp(a) decreased after 6 weeks.

### 3.3. Relationship between Serum Lipoprotein Functions and Other Clinical and Laboratory Variables

Because of the limited number of patients involved in this study, regression analyses were not performed in the PsA and AS subgroups but considered all SpA patients for which the complete dataset for the three time points was available (*n* = 51, of which PsA = 31, AS = 20). For multivariable analyses, the joint association of CEC and CLC values with other parameters was adjusted for LDL-C, HDL-C, age, sex, and inflammation indexes.

### 3.4. SR-BI CEC

SR-BI CEC was consistently, positively related to HDL-C, both at baseline, 6 weeks, 6 months, and when considering changes with treatment (*p* < 0.005 in all cases; data on changes not shown). The relationship of SR-BI with HDL-C remained significant when adjusting for age, sex, inflammation, and LDL-C at all time points, as well as when considering changes of the two parameters between baseline and 6 weeks, and between baseline and 6 months (Table 4). A positive correlation was found between coffee intake and SR-BI CEC at all time points (*p* = 0.01, 0.02 and 0.02), and this was independent of age, sex, inflammation, HDL-C and LDL-C at baseline and at 6 months. SR-BI CEC further correlated with RHI at baseline (*p* = 0.03). Moreover, the changes of SR-BI CEC and those of ESR from baseline to 6 weeks and to 6 months were negatively related (*p* = 0.04 and 0.02, respectively); the latter association persisted after adjustment for the above variables (Table 5).

### 3.5. ABCG1 CEC

ABCG1 showed positive association with HDL-C at 6 months (*p* = 0.046) and negative association with HbA1c at baseline and at 6 weeks (*p* = 0.01 and 0.03); the association with HbA1c was present at baseline also when adjusting for age, sex, inflammation, LDL-C and HDL-C (Table 4). ABCG1 was also positively associated with coffee intake at 6 weeks (*p* = 0.04) and showed a tendency towards association also at 6 months (*p* = 0.06). Similar associations were also observed at 6 weeks and 6 months when applying the above adjustments (Table 4). Moreover, the changes of ABCG1 CEC and those of complement activation (in terms of sC5b-9) from baseline to 6 weeks were negatively related, also when adjusting for the other variables (Table 5). The changes of ABCG1 CEC were negatively related to those of ESR (*p* = 0.05) and CRP (*p* = 0.01) after 6 months.

### 3.6. ABCA1 CEC

ABCA1 was positively associated with LDL-C at baseline and at 6 months (*p* < 0.005 in both cases), also when adjusting for age, sex, inflammation, and HDL-C (Table 4). A similar association was found with TG at baseline and 6 months (*p* = 0.02 and <0.005), persistent at baseline with adjustment for the above parameters and for LDL-C (Table 4). Furthermore, changes in ABCA1 with treatment from baseline to 6 months were significantly, positively associated with changes in triglycerides (*p*< 0.005), both in crude and adjusted analyses (Table 5).

### 3.7. CLC

CLC was positively associated with LDL-C at baseline and 6 weeks (*p* = 0.02 and 0.01, respectively) independently of age, sex, inflammation, and HDL-C. Correspondingly, changes in CLC from baseline to 6 months were associated with changes in LDL-C, independently of the above parameters (Table 5). Reduced CLC from baseline to 6 weeks was significantly associated with improved RHI in univariate analyses (*p* = 0.04); when adjusting for age, sex, inflammation, HDL-C and LDL-C, the association was close to statistically significant (*p* = 0.06, results not shown).

## 4. Discussion

In this study, we focused on the association between post-therapy modification of serum lipoprotein function and CVD determinants in a population of PsA and AS patients. In fact, patients with SpA are affected by accelerated atherosclerosis and increased CV risk, due to various traditional and disease-specific factors [1,2,3,33]. In SpA, as in other rheumatologic diseases, treatment is usually associated with an increase in proatherogenic lipoprotein serum levels, an aspect that raises some concern about its impact on CV risk. Nonetheless, the atherosclerotic process is highly influenced not only by the serum lipoprotein levels, but also by their quality, including for example the anti-atherogenic ability of HDL to promote cell cholesterol efflux. Indeed, a majority of the studies available so far point to an inverse correlation between HDL CEC and CVD risk [34].

Considering our SpA patients as a whole, treatment was not associated with changes in functional properties of circulating lipoproteins—CEC and CLC—despite significant modifications of serum lipid profile. In fact, after therapy, we found an increase in TC, HDL-C, apoA1 and a reduction in Lp(a) concentration, findings that are in accordance with previous studies [35,36]. As known, CEC and CLC do not merely reflect circulating lipoprotein levels, and indeed their value consists in their capacity to measure the functional impact of lipoproteins on cell cholesterol homeostasis. As PsA and AS showed significant differences both in serum lipid profile at baseline and following treatment, we analyzed the data relative to the two diseases separately.

In PsA, baseline TC was higher than in AS. This was probably due to statin use in about 30% of AS patients, as this difference was no longer apparent after adjusting for statin use. Although statins might affect baseline lipid differences in our study cohort, doses were kept stable during the study, so changes in lipid parameters after anti-rheumatic treatment were likely not affected by these drugs. Notwithstanding the small numbers of patients in both groups, we found significant differences in the effects of treatment in PsA and AS. In PsA, serum lipid profile modifications included a significant reduction of Lp(a) after 6 weeks and an increase in HDL-C and apoA1 after 6 months, effects potentially beneficial in terms of CV risk. No differences were observed with respect to CLC and CEC, so in our small cohort, antirheumatic treatment appeared not to impact on cell cholesterol-related lipoprotein functions in this group.

In AS, serum lipid profile was more profoundly affected by therapy, with a significant increase in TC, HDL-C and apoA1, and a decrease in Lp(a). With respect to lipoprotein function, CLC was unchanged, but SR-BI mediated CEC was significantly increased after 6 weeks of therapy. These results are quite relevant, as the lack of increase in CLC in presence of sustained raise of TC and LDL-C stands against a pro-atherogenic effect of treatment and adds information on the debated issue of proatherogenic lipoprotein increase after treatment. The lack of increase in serum CLC might be due to a concomitant improvement of LDL quality and to decreased Lp(a) levels. In fact, Lp(a) seems to favor cell cholesterol internalization through a non-regulated receptor pathway [37].

Lp(a) was reduced in all patients throughout the study. This is important, as high levels of this lipoprotein is an established risk factor for CVD [38]. In fact, Lp(a) tends to be increased in autoimmune diseases, and while Lp(a) levels are thought to be mainly genetically determined, several studies have shown that anti-inflammatory treatment, such as TNFi and Tocilizumab, reduces Lp(a) in patients with chronic inflammation [23,39]. These increased levels of Lp(a) in autoimmune conditions might be explained by the fact that inflammatory cytokines, for instance IL-6, induces Lp(a) production in hepatocytes [40].

The increase we observed in SR-BI CEC in parallel with serum HDL-C and apoA1 increase is not trivial, considered that in some cases increased HDL-C levels is not mirrored by an improvement in HDL protective activity [41]. Thus, given the important positive effects of SR-BI CEC on vessels [42,43], anti-TNF treatment in AS seems beneficial in terms of cell cholesterol-related lipoprotein functions and atheroprotection.

Several associations of CEC and CLC with various other laboratory and instrumental parameters that we found add information to the issue of lipoprotein function modifications upon treatment in SpA. The relationship between SR-BI CEC with serum HDL-C and ApoA1 at all time points is consistent with the concept that the large majority of circulating HDL are mature particles, which accept cholesterol effluxed via the SR-BI transporter [44]. A similar explanation for the less robust, but detectable, relationship for ABCG1 CEC might be proposed.

More complex is the issue of the possible mechanisms underlying the direct correlation between ABCA1 CEC and TC, LDL-C, non-HDL-C, and TG. Such findings might be explained by the interplay between HDL and LDL or VLDL and cholesteryl ester transfer protein (CETP) in serum [45]. This enzyme transfers esterified cholesterol from HDL to apoB-containing lipoproteins in exchange for TG, thus promoting the generation of new, lipid poor HDL particles or free ApoAI. These act as acceptors for ABCA1-mediated efflux, thus reducing the number of more mature particle specific for ABCG1-CEC. This effect is probably not quantitatively relevant as to impact on the absolute ABCA1 CEC values but might be sufficient to induce respectively a direct or inverse relationship of ABCA1 and ABCG1 CEC with proatherogenic lipoproteins that are substrate of CETP. Similarly, the strong relationship of ABCA1 CEC with TG levels might be mediated by CETP activity, as previously reported in diabetic patients [46,47].

We can exclude a role for LDL as a direct promoter of ABCA1 mediated cell cholesterol efflux, that has been described previously [48], because we used apoB-depleted serum in our experiments. Indeed, apoB-depletion through PEG precipitation has recently been confirmed to strictly reflect HDL activity [49].

The direct relationship between SR-BI CEC and coffee intake at all time points, detected also for ABCG1 CEC after treatment, is a novel finding, indicating that coffee might elicit a positive effect on cell cholesterol related lipoprotein functions, especially relative to mature HDL particles. This relationship was independent of HDL-C at baseline, and dependent on HDL-C at 6 weeks and 6 months. Our finding suggests that coffee consumption correlates with HDL molecules that are indeed able to promote cell cholesterol efflux; a finding in line with previous research demonstrating that coffee increases HDL efflux and protein levels of ABCG1 and SR-BI [50].

The positive correlation of SR-BI CEC with RHI at baseline, absent after adjusting for HDL-C serum levels, might be related to the known protective effect of HDL on endothelial cells [42,51], possibly through the interaction with cell SR-BI [52]. However, the increase observed in SR-BI CEC following treatment (statistically significant in AS patients) was not associated with a parallel improvement of RHI. This discrepancy might be due to different effects of different medications or reflect a false positive finding at baseline. Actually, in a previous observation of ours, anti-TNF treatment was associated with a less favorable effect on endothelial function than MTX alone [53]. Considering that a cross-sectional study [54] reports better RHI values in rheumatoid arthritis patients treated with TNFi compared to those treated with MTX, further studies to clarify the effect of different treatments on endothelial function in different rheumatologic diseases might be warranted.

The changes of SR-BI CEC and ABCG1 CEC with treatment were inversely related to inflammations indices (ESR, CRP and sC5b-9, a marker of complement activation), consistent with the known link between inflammation and cell cholesterol-related HDL functions, reported also in the general population [55]. These novel results are in line with research linking inflammation to reduced CEC, possibly due to modifications in HDL composition [17,23,24,56,57,58]. However, some conflicting data have been reported in the literature. For example, one study showed that the acute phase protein serum amyloid A can promote cholesterol efflux both in free form and when carried by HDL [59]. Like several other studies, we found no link between baseline CRP or ESR and CEC [10,25,60].

We also found a negative correlation between ABCG1 CEC and HbA1c both before and after therapy. This is in line with previous research reporting an inverse relationship between ABCG1 CEC and serum glucose levels. We hypothesize that this finding might be related to the effect of serum glucose on oxidative processes and glycosylation of HDL components [61].

CLC changes with treatment were inversely related to RHI changes, indicating that changes in the quality of proatherogenic lipoproteins impacted on their capacity to increase cell cholesterol content as well as endothelial function [62,63], although the underlying pathophysiological mechanisms have yet to be clarified. However, cell cholesterol content and endothelial function are indeed related, particularly in an inflammatory state, such as in SpA patients before treatment: cytokines such as TNF and IL-6 induce increase oxidation [64], as well as expression of scavenger receptors (SR-A and LOX-1), resulting in intracellular accumulation of oxLDL [65]. As accumulation of intracellular cholesterol or oxLDL can also reduce eNOS activity, this could impair endothelial function [63,66,67,68].

Our study is limited by the inherent shortcomings of observational studies, preventing any firm conclusions about treatment effects. Nevertheless, we sought to control for potential confounders through regression analyses, and we acknowledge that our observations necessitate confirmation in larger, preferably randomized controlled trials. Other limitations of our study, performed in a real-life situation, are mainly related to the small number of patients, increasing the risk of both type 2 errors results and not allowing for sub-analyses with respect to the various drugs used. In addition, the skewed gender balance in AS patients might affect the results observed both in lipid levels and other parameters. Further, the lack of the associations might be false due to low power for certain analyses. Moreover, different TNFi were used, possibly confounding the results, as differences in the effects of single drugs on lipid metabolism have been described [69]. Finally, regression analyses were performed on the whole SpA population, possibly masking disease-specific effects. However, our study has the advantage to reflect real life situation and to enrich the scant available information on lipoprotein functions and their modifications upon treatment in PsA and AS. Indeed, we provide novel pre- and post-treatment data adding to previous cross-sectional studies.

In this study, we provide novel data suggesting that anti-rheumatic treatment might be associated with a favorable modulation of overall lipoprotein function in SpA patients, particularly in AS. In spite of total and LDL serum cholesterol increase, CLC was unchanged, while HDL serum cholesterol increase was paralleled by an increase in SR-BI CEC. We also provide novel clues on possible links between lipoprotein quality and metabolism, inflammation, and vessel function, that might be further explored and validated in future investigations.

## Figures and Tables

**Table 1:** Baseline characteristics of the two patient groups.

	PsA (*n* = 37)	AS (*n* = 25)	*p* Value
Demographics			
Age, years; mean (SD)	51 (13)	51 (11)	0.90
Men (sex); *n* (%)	19 (51)	20 (80)	**0.03**
Treatment regime:			
Anti-TNF; *n* (%)	9 (24)	25 (100)	**<0.001**
MTX; *n* (%)	18 (49)	0 (0)	**<0.001**
Anti-TNF + MTX; *n* (%)	10 (27)	0 (0)	**<0.001**
Inflammatory markers			
sC5b-9 (CAU/mL); median (IQR)	0.7 (0.3)	0.9 (0.6)	0.07
WBC (10^9^/L); median (IQR)	6.3 (3)	7.7 (2)	0.09
ESR (mm/h); median (IQR)	7.5 (13)	12 (10)	0.26
CRP (mg/L); median (IQR)	5.0 (8)	10 (12)	0.14
CV risk factors			
RHI; median (IQR)	2.0 (0.7)	1.8 (0.7)	0.57
ED; *n* (%)	13 (36)	9 (36)	>0.99
BMI; median (IQR)	26 (7)	27 (6)	0.55
Current smoker; *n* (%)	8 (22)	12 (48)	**0.047**
Hypertension; *n* (%)	12 (32)	8 (32)	>0.99
HbA1C; median (IQR)	5.6 (0.5)	5.5 (0.4)	0.68
Established CVD; *n* (%)	2 (5)	5 (20)	0.11
Disease activity			
BASMI; mean (SD)	NA	3.8 (2.7)	-
BASDAI; mean (SD)	4.8 (2.2)	5.5 (2.3)	0.25
BASFI; mean (SD)	35 (22)	41 (19)	0.28
MHAQ; median (IQR)	0.4 (0.6)	0.4 (0.4)	0.16
Co-medication			
Statin; *n* (%)	2 (5)	8 (32)	**0.01**
Acetylsalicylic acid; *n* (%)	2 (5)	3 (12)	0.38
NSAIDS; *n* (%)	20 (54)	17 (68)	0.30
ACE-inhibitor/AT2; *n* (%)	4 (11)	4 (16)	0.70
Glucocorticoids; *n* (%)	5 (13)	3 (12)	>0.99
CCB; *n* (%)	2 (7)	3 (12)	0.65
Serum lipid profile			
Total cholesterol (mmol/L); mean (SD)	5.4 (0.7)	4.9 (1.2)	**0.02**
LDL-C (mmol/L); mean (SD)	3.5 (0.6)	3.1 (1.0)	0.08
HDL-C (mmol/L); median (IQR)	1.4 (0.5)	1.2 (0.4)	0.19
Triglycerides(mmol/L); median (IQR)	1.2 (0.8)	1.3 (0.7)	0.43
Lp(a) (mg/L); median (IQR)	98 (366)	96 (286)	0.95
Non-HDL-C (mmol/L); mean (SD)	3.9 (0.9)	3.6 (1.1)	0.23
ApoA1; median (IQR)	1.4 (0.3)	1.4 (0.4)	0.44
Lipoprotein function			
SR-BI CEC (%); median (IQR)	3.3 (2.1)	3.4 (1.7)	0.88
ABCG1 CEC (%); median (IQR)	4.7 (1.2)	4.4 (2.7)	0.45
ABCA1 CEC (%); median (IQR)	3.0 (3.2)	2.7 (2.5)	0.27
CLC (g chol/g DNA); median (IQR)	30.2 (10)	27.5 (6.1)	**0.04**

For normally distributed variables, values are expressed as mean ± standard deviation (SD). For non-normally distributed variables, values are expressed as median and interquartile range (IQR). Significant *p* values are in bold.

**Table 2:** Functional lipid parameters and serum lipid levels in SpA patients.

SpA Patients (*n* = 62)					
	Baseline	6 Weeks		6 Months	
	Median (IQR)	Median (IQR)	*p* vs. Baseline	Median (IQR)	*p* vs. Baseline
SR-BI CEC (*n* = 51)	3.3 (1.8)	3.6 (1.6)	0.29	3.5 (1.8)	0.09
ABCG1 CEC (*n* = 48)	4.7 (1.3)	4.8 (1.4)	0.67	5. 0 (2.0)	0.91
ABCA1 CEC (*n* = 50)	2.9 (2.3)	3.2 (2.5)	0.25	3.1 (2.4)	0.71
CLC	28.9 (8.1)	28.4 (10.4)	0.36	30.4 (14.3)	0.81
TC	5.2 (1.3)	5.4 (1.4)	**0.01**	5.6 (1.7)	**0.02**
LDL	3.2 (1.3)	3.4 (1.3)	0.15	3.2 (1.5)	0.09
Lp(a)	98 (292)	76 (266)	**<0.001**	83 (243)	**0.02**
Non-HDL	3.8 (3.9)	3.9 (1.5)	**0.04**	3.6 (1.4)	0.20
HDL	1.3 (1.4)	1.4 (0.6)	**0.04**	1.4 (0.6)	**<0.001**
ApoA1	1.4 (1.6)	1.6 (0.3)	**<0.001**	1.5 (0.3)	**<0.001**
Triglycerides	1.2 (1.2)	1.2 (0.9)	0.30	1.2 (0.7)	0.78

Values are expressed as median and interquartile range (IQR). Significant *p*-values comparing post-treatment to baseline results are in bold. The number of patients with all three time points values available for CEC and CLC are indicated for each parameter. Lipid profile for all three time points was available in 48 patients.

**Table 3:** Functional lipid parameters and serum lipid levels in PsA and AS.

PsA (*n* = 37)					
	Baseline	6 Weeks		6 Months	
	Median (IQR)	Median (IQR)	*p* vs. Baseline	Median (IQR)	*p* vs. Baseline
SR-BI (*n* = 30)	3.3 (2.1)	3.4 (2.0)	0.62	3.5 (1.7)	0.41
ABCG1 (*n* = 28)	4.7 (1.2)	5.8 (1.2)	0.13	4.7 (1.1)	0.81
ABCA1 (*n* = 30)	3.0 (3.2)	3.0 (2.7)	0.66	3.2 (2.4)	0.98
CLC (*n* = 27)	30.2 (10.3)	29.0 (10.8)	0.92	34.7 (14.6)	0.65
TC	5.3 (0.7)	5.7 (1.1)	0.22	5.6 (1.7)	0.41
LDL-C	3.4 (0.6)	3.6 (1.3)	0.81	3.7 (1.6)	0.52
Lp(a)	98 (366)	78 (519)	**<0.001**	78 (562)	0.12
Non-HDL-C	3.9 (0.9)	4.2 (1.4)	0.24	3.9 (1.7)	0.52
HDL-C	1.3 (0.5)	1.4 (0.6)	0.99	1.4 (0.6)	**0.03**
ApoA1	1.4 (0.3)	1.5 (0.3)	**0.049**	1.5 (0.4)	**0.01**
Triglycerides	1.0 (0.8)	1.2 (1.1)	0.22	1.2 (0.8)	0.49
**AS (*n* = 25)**					
	**Baseline**	**6 Weeks**		**6 Months**	
	**Median** **(IQR)**	**Median** **(IQR)**	***p* vs. Baseline**	**Median** **(IQR)**	***p* vs. Baseline**
SR-BI (*n* = 21)	3.4 (1.7)	4.9 (2.2)	**0.03**	3.5 (2.1)	0.10
ABCG1 (*n* = 20)	4.4 (2.7)	4.8 (2.7)	0.29	5.4 (2.1)	0.84
ABCA1 (*n* = 20)	2.7 (2.5)	3.5 (2.0)	0.16	3.1 (2.3)	0.55
CLC	27.5 (6.1)	26.4 (9.4)	0.16	28.5 (7.6)	0.97
TC	5.0 (1.8)	5.3 (1.8)	**<0.001**	5.2 (2.1)	**0.01**
LDL-C	2.8 (1.2)	3.1 (1.3)	**0.02**	3.1 (1.6)	0.05
Lp(a)	96 (271)	77 (228)	**0.02**	88 (208)	0.09
Non-HDL-C	3.2 (1.6)	3.5 (1.5)	0.06	3.5 (1.5)	0.21
HDL-C	1.3 (0.3)	1.4 (0.6)	**0.001**	1.4 (0.6)	**0.01**
ApoA1	1.4 (0.3)	1.6 (0.3)	**<0.001**	1.5 (0.3)	**0.03**
Triglycerides	1.3 (0.7)	1.3 (0.7)	0.97	1.2 (0.6)	0.23

Values are expressed as median and interquartile range (IQR). Significant *p* values comparing post-treatment to baseline results are in bold. The number of patients with all three time points values available for CEC and CLC are indicated for each parameter. Lipid profile for all three time points was available in 28 patients with PsA and 20 with AS.

**Table 4:** Adjusted regression analyses: Variables with a statistically significant association to cholesterol transport parameters at baseline, 6 weeks, and at 6 months.

SR-BI	Baseline				6 Weeks			6 Months		
	B (95% CI)			*p*	B	95% CI	*p*	B	95% CI	*p*
HDL-C	1.68 (0.91 to 2.45)			<0.005	1.92	1.27 to 2.57	<0.005	1.67	0.94 to 2.40	<0.005
Coffee	0.72 (0.19 to 1.25)			0.01				0.53	0.00 to 1.05	0.049
**ACBA1**	**Baseline**				**6 Weeks**			**6 Months**		
	**B (95% CI)**			***p***	**B**	**95% CI**	***p***	**B**	**95% CI**	***p***
LDL-C	1.07 (0.55 to 1.58)			<0.005				0.82	0.28 to 1.36	<0.005
TRG	1.16 (0.33 to 1.99)			0.01						
**ABCG1**	**Baseline**				**6 Weeks**			**6 Months**		
	**B (95% CI)**			***p***	**B**	**95% CI**	***p***	**B**	**95% CI**	***p***
HDL-C								0.81	0.02 to 1.61	0.046
Coffee					1.01	0.27 to 1.75	0.01	0.65	0.02 to 1.28	0.04
HbA1c	0.90 (−1.82 to 0.01)			0.05						
**CLC**	**Baseline**				**6 Weeks**			**6 Months**		
	**B (95% CI)**			***p***	**B**	**95% CI**	***p***	**B**	**95% CI**	***p***
LDL-C	3.37 (0.62 to 6.12)			0.02	3.98	0.76 to 7.19	0.02			

All variables shown have been adjusted for age, sex, HDL-C, LDL-C, CRP and ESR. Abbreviations: B: estimated regression coefficient, *p*: *p*-value for the null hypothesis of a zero regression coefficient, TRG: triglycerides, ESR: erythrocyte sedimentation rate, CRP: C-reactive protein, HDL-C: high-density lipoprotein cholesterol, LDL-C: low-density lipoprotein cholesterol; HbA1c: Hemoglobin A1c.

**Table 5:** Adjusted regression analyses: Variables with a statistically significant association to cholesterol transport parameters for changes from baseline to 6 weeks, and from baseline to 6 months.

SR-BI	Change from Baseline to 6 Weeks		Change from Baseline to 6 Months		
	B (95% CI)	*p*	B	95% CI	*p*
HDL-C Δ	1.66 (80.86 to 2.46)	<0.005	2.46	1.46 to 3.46	<0.005
ESR Δ			−0.04	0.08 to 0.01	0.01
**ACBA1**	**Change from Baseline to 6 Weeks**		**Change from Baseline to 6 Months**		
	**B (95% CI)**	***p***	**B**	**95% CI**	***p***
TRG Δ			0.82	0.16 to 1.48	0.02
**ABCG1**	**Change from Baseline to 6 Weeks**		**Change from Baseline to 6 Months**		
	**B (95% CI)**	***p***	**B**	**95% CI**	***p***
sC5b-9 Δ	−1.22 (−2.37 to −0.08)	0.04			
**CLC**	**Change from Baseline to 6 Weeks**		**Change from Baseline to 6 Months**		
	**B (95% CI)**	***p***	**B**	**95% CI**	***p***
LDL-C Δ			−3.53	−7.13 to 0.06	0.05

All variables shown have been adjusted for age, sex, HDL-C, LDL-C, CRP and ESR. Δ indicates change from baseline to 6 weeks/6 months. Abbreviations: B: estimated regression coefficient, *p*: *p*-value for the null hypothesis of a zero regression coefficient, RHI: reactive hyperemia index, TRG: triglycerides, ESR: erythrocyte sedimentation rate, CRP: C-reactive protein, HDL-C: high-density lipoprotein cholesterol, LDL-C: low-density lipoprotein cholesterol; cC5b-9: soluble complement C5b-9.

## Data Availability

The data presented in this study are openly available in FigShare at doi:10.6084/m9.figshare.20967475.
